# What do young people really understand when completing questionnaires? Lessons learnt from developing a questionnaire to measure behavioural outcomes in a sexual health trial

**DOI:** 10.1186/1745-6215-16-S2-P79

**Published:** 2015-11-16

**Authors:** Lisa Maguire, Aine Aventin, Maria Lohan, Mike Clarke

**Affiliations:** 1 Queen's University Belfast, Belfast, UK; 2 University of Liverpool, Liverpool, UK

## 

It cannot be taken for granted that young people will understand and interpret questions in the way we expect. This is true even when using validated measures, developed and used with the appropriate age group. It can cause problems for trials that need to minimise “noise” when measuring outcomes. We will describe our observations and lessons learnt from developing a questionnaire for young people in a sexual health trial. The *Jack* feasibility trial, funded by the NIHR evaluates a film-based educational intervention aimed at increasing boys’ and girls’ intentions to avoid teenage pregnancy (ISRCTN11632300). The trial is ongoing and has recruited eight post-primary schools and 831 young people. As part of the trial, we compiled validated and non-validated instruments to measure sexual behavior data including engagement in sexual intercourse, contraception use, drugs and alcohol usage, diagnosis of STIs; data regarding knowledge, attitudes, skills and intentions relating to avoiding teenage pregnancy; and socio-economic status. The questionnaire was piloted and appropriately modified after pupils took part in focus groups to identify procedural challenges in completing the questionnaire. We discovered difficulties in questionnaire completion due to understanding of terminology and ensuring privacy in different classroom layouts. We will also discuss the issues young people raised when completing the questionnaire, for example by examining the challenges encountered with specific questions such as ‘Have you ever drunk alcohol?’ and present a qualitative description of issues surrounding understanding. We will highlight the importance of feasibility and pilot work for school-based trials in sexual health.

